# Ecosystem services and disservices in the Luanhe River Basin in China under past, current and future land uses: implications for the sustainable development goals

**DOI:** 10.1007/s11625-021-01078-8

**Published:** 2022-01-08

**Authors:** Jiren Xu, Brian Barrett, Fabrice G. Renaud

**Affiliations:** 1 School of Interdisciplinary Studies, University of Glasgow, Dumfries, UK; 2 School of Geographical and Earth Sciences, University of Glasgow, Glasgow, UK

**Keywords:** Ecosystem services, Ecosystem disservices, Luanhe river basin, Sustainable development goals

## Abstract

Understanding how ecosystem services (ES) and ecosystem disservices (EDS) are affected by human-induced landscape changes is important to minimise trade-offs and maximise synergies between Sustainable Development Goals (SDGs) and targets, and for equitable development across governance scales. However, limited research investigates how ES and EDS can change under past, current, and future land uses. This study, conducted in the Luanhe River Basin (LRB), demonstrates the interaction between humans and the environment under past, current, and future land uses at the river basin scale in China, using a stakeholders’ participatory capacity matrix to characterise both ES and EDS. Results indicate that forests and water bodies provided the highest overall ES capacity, while the lowest scores were reached in built-up and unused land areas. Built-up land and cropland provided the highest overall EDS, while the lowest EDS scores were for water bodies. By applying the ecosystem services potential index (ESPI) and ecosystem disservices potential index (EDSPI), we found that the ESPI of all the ES declined from 1980 to 2018 and would continue to decline until 2030 without sustainable and conservation development strategies in the LRB. The EDSPI under all future scenarios in 2030 was projected to increase compared to the baseline in 1980. This study recommends establishing and implementing sustainable environmental protection policies and cross-regional and trans-provincial eco-compensation schemes for minimising trade-offs in ES. The study proposes an integrated research framework that could be useful for understanding the effect of historical and future human–environment interactions on ES and EDS, and SDGs achievement.

**Supplementary Information:**

The online version contains supplementary material available at 10.1007/s11625-021-01078-8.

## Introduction

The Sustainable Development Goals (SDGs) call to end poverty, protect the planet and ensure that all people enjoy peace and prosperity by 2030 (UNDP 2015). Ecosystems and the services they provide underpin all dimensions of human, societal, cultural and economic well-being (Folke et al. 2016; Naeem et al. 2012), which are directly related to the SDGs. The concept of Ecosystem Services (ES) provides a useful framework to communicate and analyse the ecological and socioeconomic impacts of land use decisions, and all SDGs benefit to some degree from ecosystem protection, restoration, and sustainable use (ICSU 2015). Therefore, a better understanding of the ES and integrating ES into strategies for meeting the SDGs is essential (Johnson et al. 2019; Wood et al. 2018).

ES are defined and classified in various ways, for example, by the Millennium Ecosystem Assessment (Millennium Ecosystem Assessment, 2005), The Economics of Ecosystems and Biodiversity (TEEB) (Kumar, 2010), and by Costanza et al. (1997), but all definitions revolve on values and benefits that ecosystems contribute to human well-being. One of the most widely used definitions is that of the Millennium Ecosystem Assessment (2005), which defined ecosystem services as benefits people obtain from ecosystems. The Millennium Ecosystem Assessment (2005) provided four major categories of ES: provisioning services (PS), regulating services (RS), cultural services (CS) and supporting services (ecological integrity: EI). However, ecosystems can also produce and deliver various goods and services that are economically or socially harmful, compromise health, or are even life-threatening. These are referred to as ecosystem disservices (EDS) and are increasingly accounted for when evaluating ecosystems (Campagne et al. 2018; Lyytimäki and Sipilä 2009; von Döhren and Haase 2015). EDS has been defined as ‘the ecosystem generated functions, processes and attributed that result in perceived or actual negative impacts on human well-being’ (Shackleton et al. 2016), which mirrors the definition of ES. Studying EDS is important to limit trade-offs between SDGs. However, compared with ES, EDS draws much less research attention: the total literature on EDS was only 0.7% of that on ES between 1976 and 2018 (Blanco et al. 2019). EDS is even absent from most recent ES conceptual advances (Costanza et al. 2017; Haines-Young and Potschin 2018). Very limited research takes EDS into account at a river basin scale (Khan et al. 2019; Sun et al. 2020).

The quantification and implementation approaches of ES and EDS vary in complexity, research scale, resource requirements and accuracy. Examples include the capacity matrix approach (Burkhard et al. 2009, 2012) based on participatory expert opinion via quantitative proxies assigned per land use type based on literature or experts; simple biophysical models (e.g. InVEST toolset, Sharp et al. (2020)); integrated models of the dynamic complexity of human-environment interactions (e.g. ARIES, Villa et al. (2009)); and resource-demanding complex process-based models (e.g. Soil and Water Assessment Tool model, Gassman et al. (2007)). Among these approaches, the expert-based capacity matrix approach of ecosystem services assessment, which considers stakeholders’ opinions, has become one of the most popular ES assessment techniques today (Jacobs et al. 2015). It is useful in providing statistical and spatial information and illustrations in environmental planning and management for guiding policymakers’ strategic planning (Burkhard et al. 2013), particularly in the context of the SDGs. The capacity matrix is a look-up table that links ecosystem types (ETs) to ES potentially provided by these ETs and allows the assessment of a higher number of ES than other assessment methods. Based on experts’ knowledge, the matrix gives a quick assessment of ES and EDS potentially provided in an area (Stoll et al. 2015; Vihervaara et al. 2012). Although this matrix has subjective limitations depending on the expert’s knowledge level and uncertainties (see Hou et al. 2013, Jacobs et al. 2015, and Campagne et al. 2017), it is a useful tool for decision-makers and environmental managers (Swetnam et al. 2011). Compared with other methods, the capacity matrix can be applied at different scales (e.g. Stoll et al. 2015; Jiang et al. 2021). It can overcome the issues of reduced quantitative data and spatial heterogeneity by asking experts to estimate scores. The capacity matrix has been widely developed and applied in case studies around the world to evaluate the ecosystem services potential or capacity (Burkhard et al. 2014, 2012; García-Llamas et al. 2019; Hermann et al. 2014; Huq et al. 2019; Jiang et al. 2021; Müller et al. 2020; Stoll et al. 2015). Furthermore, ES can be jointly assessed with EDS in the matrix, although the concept of EDS has only recently been added by Campagne et al. (2017) and Campagne and Roche (2018). The optimal expert panel size, expert confidence score, and scoring variability statistics have been proposed to address some of the criticisms on using capacity matrices (e.g. uncertainty due to the differences between experts’ knowledge) (Campagne et al. 2017).

As one of the SDGs signatories, China has published ‘China's National Plan on Implementation of the 2030 Agenda for Sustainable Development’ to stipulate detailed strategies for implementing the SDGs (P.R.C. Foreign Ministry 2016). However, with the rapid economic growth, urbanisation and industrialisation, and the growing resource consumption during the past three decades, ecological and environmental problems have increasingly become bottlenecks restricting China’s sustainable development agenda (Liu and Diamond 2005; Shapiro 2016). A river basin is a semi-closed ecological and economic system that plays an important role in global and regional development (Zhao et al. 2018). In China, river basin ecosystem degradation has decreased ES and threatens the river basin’s ecological security. Currently, there are a series of land use conflicts between ecosystem conservation, economic development, water demand and agricultural intensification (Dan et al. 2014; Fu et al. 2007; Liu and Diamond 2008; Ma 2011). Meanwhile, the ES capacity is affected by land use changes driven by current and future development scenarios. This relationship between ES and land use changes highlights the importance of ES and EDS in guiding land use planning and ecosystem management strategies to promote sustainability (Dong et al. 2018; Sun et al. 2019). Therefore, assessing ES and EDS under current and future development scenarios for China’s large river basins, especially considering stakeholders’ opinions, will help understand the human-environment interactions for achieving the SDGs in China and comparable large river basin contexts.

This study aims to evaluate the main ES and EDS, identify hotspots of critical ES and EDS, and understand the relationship between ES and EDS and river basin management in the Luanhe River Basin (LRB), a large river basin in North China, to provide policy recommendations to limit SDG trade-offs and help achieve equitable development across the river basin. Based on stakeholder interviews, field investigations and geospatial analyses, the research had five main objectives: (1) estimating ES and EDS in the LRB through the use of capacity matrices; (2) defining and mapping hotspots and coldspots of critical ES and EDS; (3) defining the ecological function zones based on the ES and EDS and regional and local policies; (4) understanding ES and EDS dynamics under past, current and future land uses; and (5) propose management measures for the different functional zones. To the best of our knowledge, this is the first research focussing on both ES and EDS using the stakeholders participatory capacity matrix for investigating the interaction of humans and the environment at the river basin scale in China.

## Materials and methods

### Study area

**Fig. 1:**
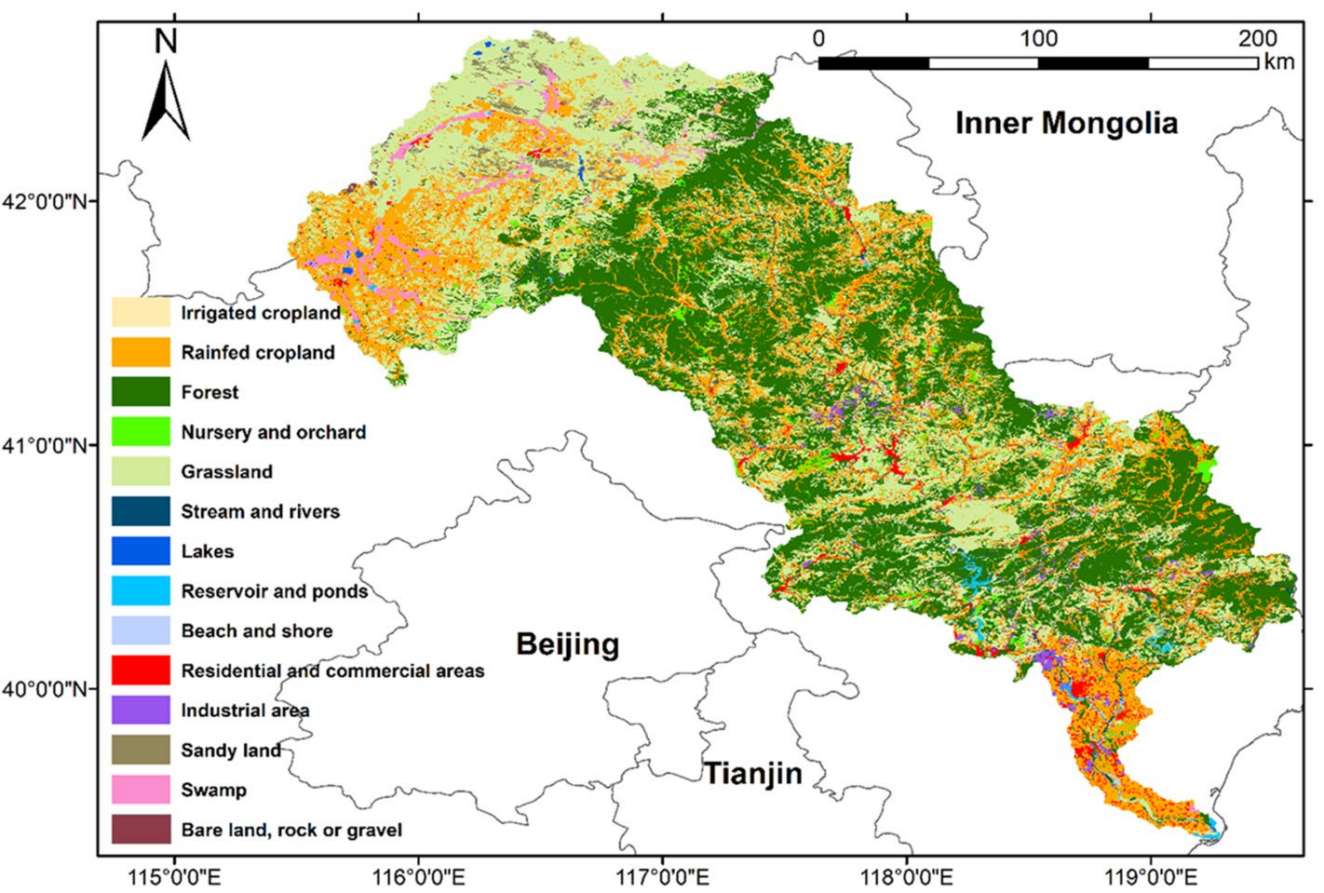
Ecosystem types of Luanhe River Basin in 2018. Land use data were acquired from China’s National Land Use and Cover Change (CNLUCC) dataset (Xu et al. 2018b) from the Resources and Environmental Sciences Data Center of the Chinese Academy of Sciences

The LRB (39°10′–42°30′ N, 115°30′–119°15′ E) is located across a semiarid region of North China (Fig. 1), with an annual average temperature and precipitation from 1982 to 2015 of 7.0 ± 2.6 °C and 488.4 ± 80.7 mm, respectively (Wu et al. 2020). It encompasses 27 counties in two provinces (Hebei and Liaoning) and one Autonomous Region (Inner Mongolia), with a total area of around 45,000 km^2^. The LRB has a population of 5.4 million, with a population density of 122 persons/km^2^ (Bi et al. 2018). The LRB is the most afforested river basin in North China, an important part of China’s biggest afforestation project since 1978—the Three-North Shelter Forest Program (Wang et al. 2010). It is an important ecological barrier to alleviate the effects of sandstorms from Mongolia on North China and an important water resource for China’s most severe water-scarce region—the Beijing–Tianjin–Hebei region (BTH) (Li et al. 2017). The predominant pastures in the north-east part of the river basin, the larger reservoirs (Panjiakou and Daheiting Reservoirs) in the middle reach, and the cropland surrounding urban areas in the south also provide multiple ES and EDS.

### ES/EDS capacity matrix

Land uses (ecosystem types, ETs) in the LRB were derived from China’s National Land Use and Cover Change (CNLUCC) dataset (Liu et al. 2014; Xu et al. 2018b) produced by the Resources and Environmental Sciences Data Center of the Chinese Academy of Sciences. The CNLUCC data for the study area were derived from Landsat TM or ETM, with a corresponding spatial resolution of 30 m × 30 m. There are six major land use and land cover categories, including cropland, forestland, grassland, water, built-up land, and unused land, which were subdivided into 25 subcategories in the CNLUCC dataset. The minimum classification accuracy of selected polygons was 94% (Xu et al. 2018b). According to the available ecosystem types of the locality and local expert knowledge, six major ETs: cropland, forest, grassland, water body, built-up land and unused land, which could be subdivided into 14 subcategories of ETs were identified for this research. Figure 1 shows the ETs of the LRB in 2018. The forests account for 37.9% of the area of the LRB, followed by grassland (31.4%), cropland (22.9%), built-up land (3.7%), unused land (2.5%), and water body (1.6%).

Based on the Millennium Ecosystem Assessment (Millennium Ecosystem Assessment 2005) and the Common International Classification of Ecosystem Services (CICES) v5.1 (Haines-Young and Potschin 2018), 11 provisioning services (PS), ten regulating services (RS), five cultural services (CS), and seven ecological integrity indicators (EI) were selected. Moreover, based on the context of local conditions, experts’ opinions, and previous studies (Campagne et al. 2018; Khan et al. 2019; Sun et al. 2020), 11 EDS were added to the matrix.

**Fig. 2:**
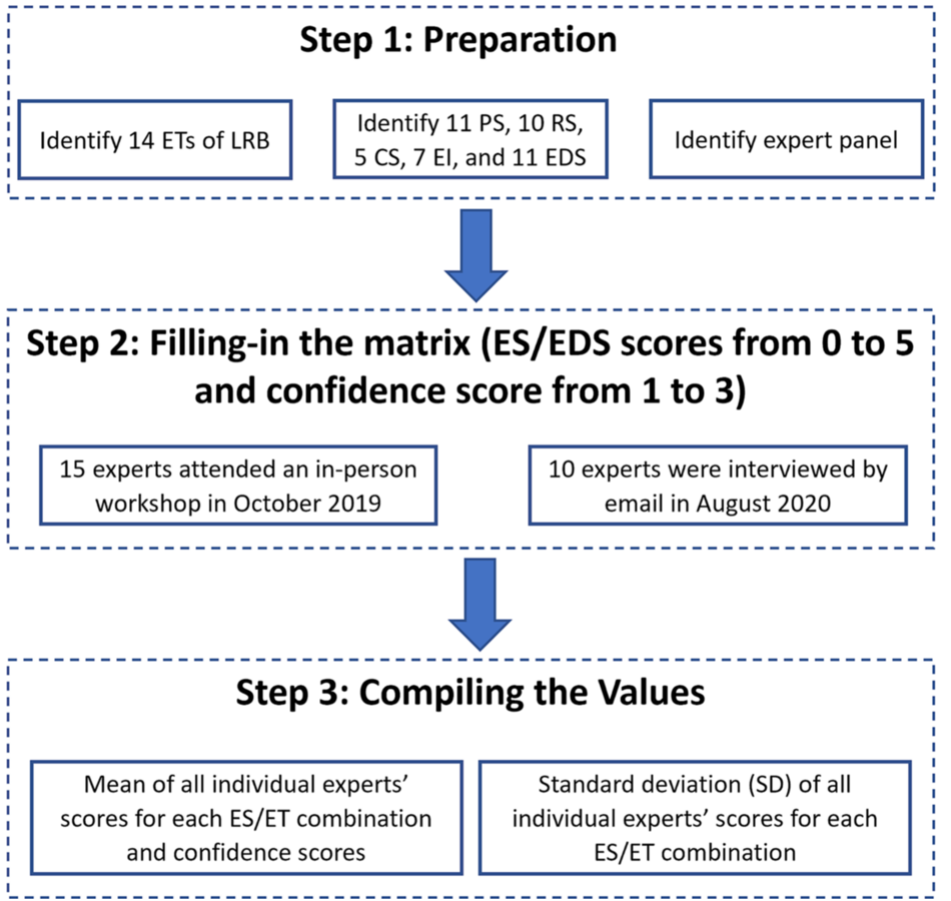
Flowchart for deriving ES/EDS matrix based on the expert knowledge approach following the guideline by Campagne et al. (2017) and Campagne and Roche (2018)

The capacity matrix was then filled following the guideline by Campagne et al. (2017) and Campagne and Roche (2018) (Fig. 2). To gather as much information as possible from a broad diversity of expertise, and evaluate score confidence levels by comparing the score variability among different experts from different backgrounds, twenty-five experts with extensive theoretical and practical knowledge of the local environment were engaged in the research during two periods. The matrix was completed in October 2019 and August 2020 by a panel of 15 experts and ten experts, respectively, based on participatory methods. Each time, experts received an explanation of the definitions and classification of ES and EDS, and were then asked to fill in the capacity matrix individually. The difference between these two sets of expert scoring exercises is that in October 2019, the experts filled the capacity matrix in-person at a workshop in Tianjin, while in August 2020, the experts filled the capacity matrix by email due to Covid-19 travel restrictions. Among these 25 experts, eight were from government bodies, 12 from research institutes, and five from regional technical bodies. Therefore, the expert panel represented diverse views from a broad range of disciplines, universities, institutions, and agencies familiar with the environmental issues and policies in the LRB.

Following Burkhard et al. (2009), ES and EDS scores were ranked from 0 to 5 to express the capacity of an ET to provide a specific ES or EDS. In addition to a score for each ES or EDS, the experts were asked to give a confidence index in their score for each ES or EDS and each ET from 1 to 3 following Campagne et al. (2017). This confidence index was used to estimate expert confidence in providing the capacity score.

The average mean score and standard deviation (SD) of ES and EDS capacities for each subcategory of ETs were produced based on the individual experts’ scores (Campagne et al. 2017; Mukherjee et al. 2018). The average mean score of the major category of ETs (i.e. cropland, forest, grassland, water body, built-up land, and unused land) was weighted by the area occupied by each subcategory of ETs among the major ETs.

There is a negative relationship between the confidence score and the mean score errors (Campagne et al. 2017). A 6-level confidence score (1, 2, 3, 4, 6, 9) for each ET and ES and EDS was obtained by multiplying three confidence levels for ETs by three confidence levels for ES and EDS. ‘Low confidence’ refers to a confidence score less than 3, ‘Moderate Confidence’ refers to a confidence score between 3 and 6, and ‘High confidence’ refers to a confidence score greater than 6.

### Mapping ES and EDS capacity matrix scores

The Jenks optimisation method (Jenks 1967), a data clustering method that has been widely used in geographic information sciences (Chen et al. 2013; Sadeghfam et al. 2016; Xu et al. 2018a), was used to classify the level of ES and EDS scores to determine hotspots and coldspots in this study. The regionalisation of ES and EDS for delineating the ecological function zones was carried out by (1) synthesising similar kinds of critical ES, EDS, ETs and physical features; (2) overlapping the hotspots of the integrated ES and EDS; (3) being characterised with major environmental problems, related ecosystems, and social driving forces correlated with management strategies (Cai et al. 2017).

### Past, current and future land use in the LRB

The past (1980) and current (2018) land use in the LRB were both derived from China’s National Land Use and Cover Change (CNLUCC) dataset (Xu et al. 2018b). The future dynamics of ETs in the LRB under different scenarios in 2030 (Table 1) was derived from the projected land systems based on the CLUMondo model (Van Asselen and Verburg 2013) by Xu et al. (2021). The land system presents the information of three main classification factors: (1) land use and cover, (2) livestock, and (3) agricultural intensity. Land use and cover represent the landscape’s composition, while livestock and agricultural intensity data represent important characteristics of land management and farming systems. Each variable’s classification threshold was arbitrarily determined by the natural breaks in the variable distribution (van Asselen and Verburg 2012). Four scenarios for the LRB in 2030: *Trend*, *Expansion*, *Sustainability*, and *Conservation* were designed based on shared socioeconomic pathways (SSPs), environmental protection targets, local plans and policies, and the information from the stakeholders’ workshop in Tianjin. The SSPs are scenarios of projected socioeconomic global changes used to derive greenhouse gas emissions scenarios with different climate policies (O’Neill et al. 2014; Riahi et al. 2017). The *Trend* scenario follows the middle-of-the-road shared socioeconomic pathway (SSP2), a pathway that does not shift markedly from historical patterns. The *Expansion* scenario follows the fossil-fuelled development shared socioeconomic pathway (SSP5), where people exploit abundant fossil fuel resources, the global economy grows at the highest speed, and the global urbanisation rate reaches 92% in 2100. The *Sustainability* scenario follows the sustainable shared socioeconomic pathway (SSP1), a sustainable pathway that is people-oriented and where land use is strongly regulated (Van Vuuren et al. 2017). The socioeconomic context of the *Sustainability* scenario was used as a baseline for the *conservation* scenario and extended by the implementation of the ecological restoration and protection policy targets. The land system simulations have been conducted in three steps: (1) calculating the relationship between the land systems and local explanatory factors for the initial year (2000); (2) parameterising and calibrating the model based on the 2015 land systems map; (3) modelling the future land system maps using the most optimised parameters. Introduction to the land system simulation workflow is in the Supplementary Text. More details on these four scenarios and how the future land systems was generated are available in Xu et al. (2021).

**Table 1:** Percentage of each land use in the LRB in 1980, 2018 and 2030 (Xu et al. 2021)

Land use	1980	2018	2030			
			Trend	Expansion	Sustainability	Conservation
Cropland	23.81	22.88	23.19	23.20	23.21	23.11
Woodland	38.56	37.93	31.58	33.32	36.43	39.88
Grassland	30.91	31.44	35.31	37.62	34.58	31.78
Water body	1.80	1.62	1.23	1.11	1.49	1.32
Built-up land	1.36	3.67	8.67	4.57	3.91	3.91
Unused land	3.56	2.46	0.02	0.19	0.39	0

### Ecosystem services potential index (ESPI) and ecosystem disservices potential index (EDSPI) under different land use changes

The Ecosystem Services Potential Index (ESPI) (Grima and Singh 2020) has been applied in this study for estimating the changes of the potential of a region to deliver ecosystem services under past, current and future land use in the LRB. The ESPI is an index to estimate a region’s potential to deliver ecosystem services based on an ecosystem service capacity matrix (Burkhard et al. 2014; Grima and Singh 2020). For applying the ESPI in this study, the 14 subcategories of ETs were merged into their corresponding major category of ETs (i.e. Cropland, Forest, Grassland, Water body, Built-up land, and Unused land) to allow for the use of the ESPI. According to Grima and Singh (2020), the ESPI value estimated can be weighted by the area occupied by each terrestrial ecosystem (including inland waters). For each major category of ETs and ES class defined in the matrix, the mean of weighted values gives output values between 0 and 5. Here, we took the Ecosystem Services Potential of Provisioning Services (ESPPS) to demonstrate how the ESPI was calculated. There are six ETs and 11 PS indicators (i.e. Crops, Livestock, Fodder, Capture fisheries, Aquaculture, Wild foods, Timber, Wood fuel, Energy (biomass), Biochemicals and medicine, and Freshwater), and each intersection can have a maximum value of 5, so the maximum Ecosystem Services Potential of Provisioning Services (ESP_PS_) possible is 330. The maximum ESP_PS_ (330) divided by the actual ESP (sum of all area occupied-weighted estimated values between 0 and 5) is the ESPI_PS_ of the LRB (dimensionless [0–1]). This method is also applied to calculate the Ecosystem Services Potential of Regulating Services (ESP_RS_), Ecosystem Services Potential of Cultural Services (ESP_CS_) and Ecosystem Services Potential of Ecological Integrity (ESP_EI_).

Similar to the ESPI, we developed the Ecosystem Disservices Potential Index (EDSPI), which estimates the potential of a region to deliver ecosystem disservices based on the score of EDS from the capacity matrix. In terms of applying the EDSPI in this study, there are six major categories of ETs and 11 EDS indicators. The maximum ecosystem disservices potential (EDSP) (330) divided by the actual EDSP (sum of all area occupied-weighted estimated values between 0 and 5) is the EDSPI of the LRB (dimensionless [0–1]).

## Results

### Capacity matrix scores

The ES and EDS capacity matrix is shown in Table 2. The matrix rows have six major categories of ETs and 14 subcategories of ETs, and the columns had 33 different ES indicators and 11 EDS indicators. Overall, the forests, lakes, and reservoirs provide the highest ES capacity, while the lowest scores were assigned to built-up and unused lands. The built-up land and cropland provide the highest overall EDS capacity, while the lowest EDS scores were assigned to water bodies.

**Table 2:**
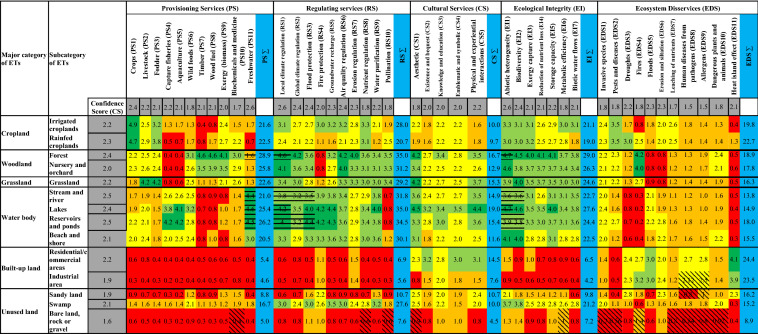
Capacity matrix with mean ES and EDS capacity scores. Mean confidence scores for ET and ES/EDS are on the table margins (grey colour cells). The values/colours of mean ES and EDS capacity scores indicate the following capacities: 0–1 = very low relevant capacity (red); 1–2 = low relevant capacity (orange); 2–3 = medium capacity (yellow); 3–4 = high capacity (light green); 4–5 = very high relevant capacity (dark green). The textures of each cell are based on multiplying each ET and ES score confidence scores with the following confidence levels: ‘Low confidence’ (diagonal) refers to a confidence score less than 3, ‘Moderate Confidence’ (no texture) refers to a confidence score between 3 and 6, ‘High confidence’(horizontal) refers to a confidence score greater than 6. ES = Ecosystem Service; EDS = Ecosystem disservices, ET = Ecosystem Type. Sums of average scores for PS, RS, CS, EI and EDS are shown in the blue columns

Forests not only occupy the largest area of the LRB (37.9%) but are also considered to be particularly important for almost all ES: PS, RS, and EI received the highest score, and CS received the second-highest score. Forests are the most important sources of timber, wood fuel, wild food and biomass energy (PS), local and global climate regulation, air quality regulation, erosion regulation, and nutrient regulation (RS). Moreover, forests were considered to have the highest ESP of EI for abiotic heterogeneity, biodiversity, exergy capture, reduction of nutrient loss, and storage capacity. However, forests are also considered to have the highest EDS for fires.

Grassland makes up the second largest part (31.4%) of the LRB. It was ranked the highest in providing for livestock and fodder. Grasslands are also considered to have the capacity to enhance biodiversity and contribute to the aesthetic value of the LRB. Cropland makes up the third-largest part (22.9%) of the LRB and has the highest score in its capacity to produce crops and generate pests and diseases. Although the lakes and reservoirs cover only 0.5% of the LRB’s area, they are important for PS, RS, CS and EI. Particularly, they have the highest score in their capacity for capture fisheries, aquaculture, and freshwater (PS), flood protection, fire protection, groundwater recharge and water purification (RS); aesthetic, emblematic and symbolic, and physical and experiential interactions (CS); abiotic heterogeneity, biodiversity (EI). The built-up lands and unused land did not have high ES capacity; however, the built-up lands have the highest EDS scores for fire, flood and heat island effects.

### Spatial pattern of ES and EDS and derived ecological function zone

**Fig. 3:**
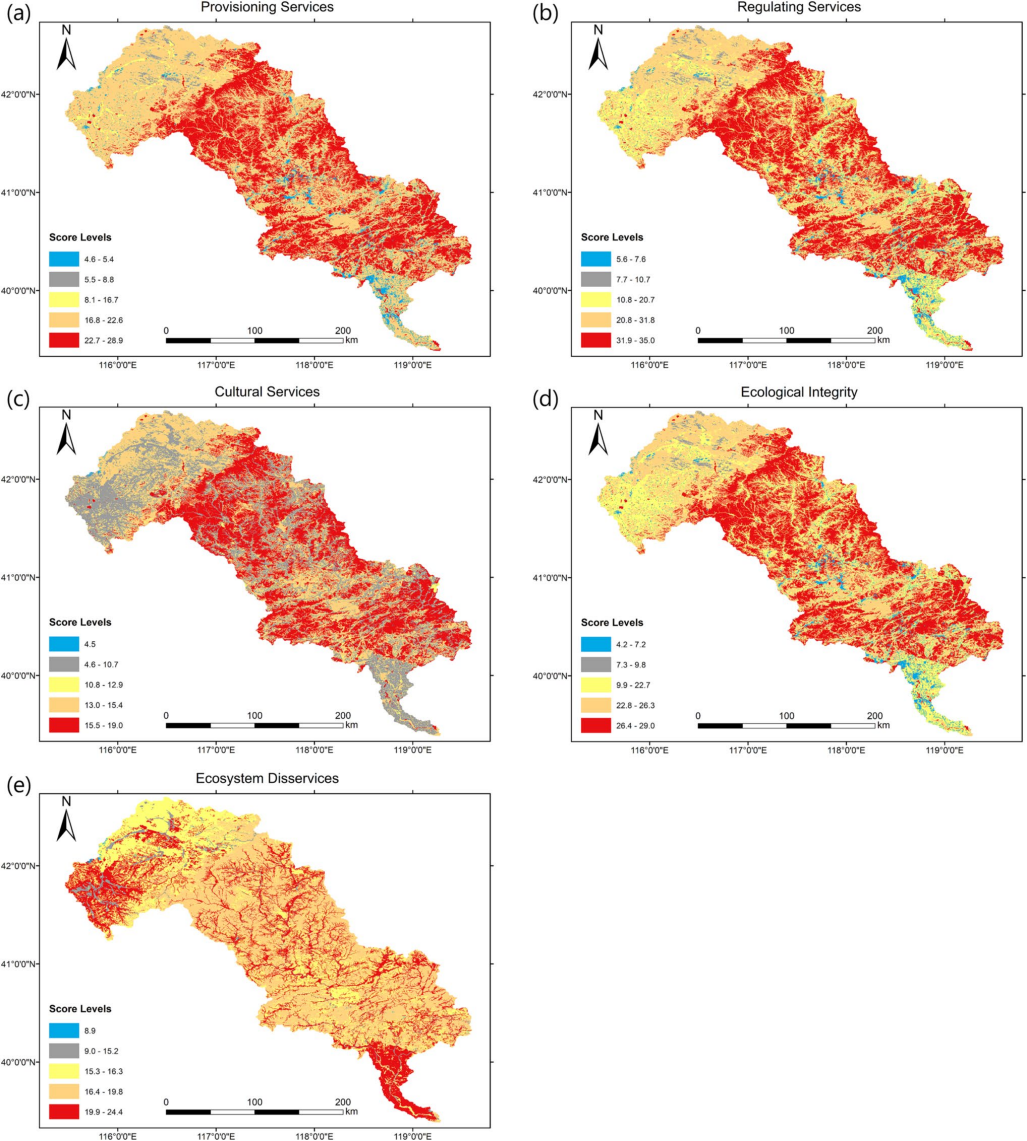
Spatial distribution of score levels of provisioning services, regulating services, cultural services, ecological integrity and ecosystem disservices in the LRB

Figure 3 shows the spatial distribution of PS, RS, CS, EI and EDS scores in the LRB. For PS, hotspots are found extensively in the upper-middle reach, including forests and orchards and the water bodies, including lakes and reservoirs.

**Fig. 4:**
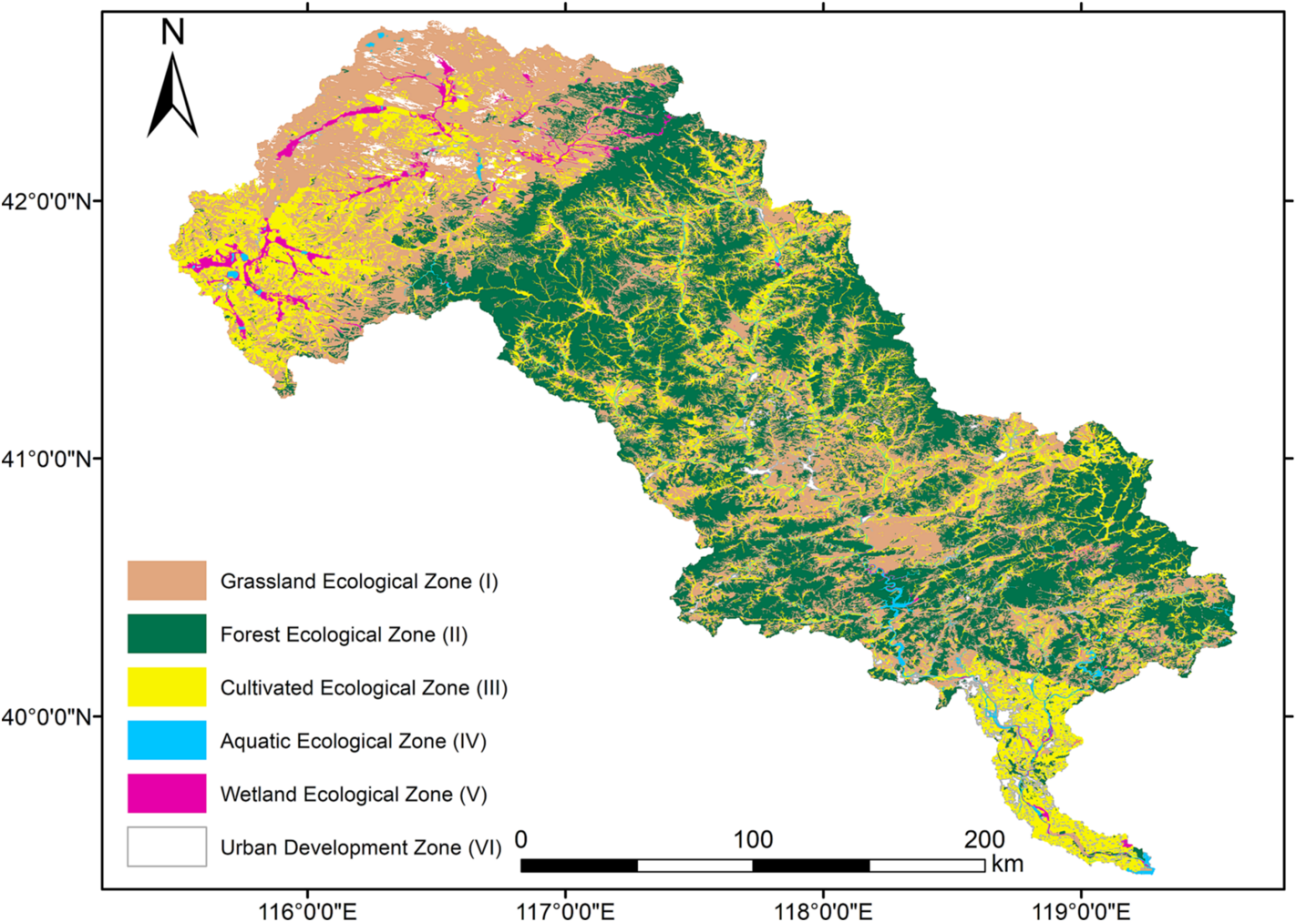
Overlap map of the integrated ES and EDS in the LRB. I: Grassland Ecological Zone, II: Forest Ecological Zone, III: Cultivated Ecological Zone, IV: Aquatic Ecological Zone, V: Wetland Ecological Zone, VI: Urban Development Zone

Coldspots were distributed in the bare land, and built-up land areas mainly concentrated in the downstream area of the basin. For RS, hotspots are also widespread in the upper-middle reach, including forests, lakes, and reservoirs, while coldspots were also distributed in the bare land and built-up land areas. For CS, hotspots were widespread in the forests in the upper-middle reach and around the lakes. Coldspots were located around bare land. For EI, hotspots were concentrated in the forests and lakes, while coldspots were distributed in the built-up land areas and the bare land. For EDS, hotspots were concentrated in the built-up land areas and the croplands. The regionalisation of critical ES and EDS was carried out by overlapping the hotspots of the integrated ES and EDS (Fig. 4) and defining six ecological functional zones (Table S1). Details for the introductions to these six ecological functional zones are in the Supplementary Text.

### ESPI and EDSPI dynamics under past, current and future land use

**Fig. 5:**
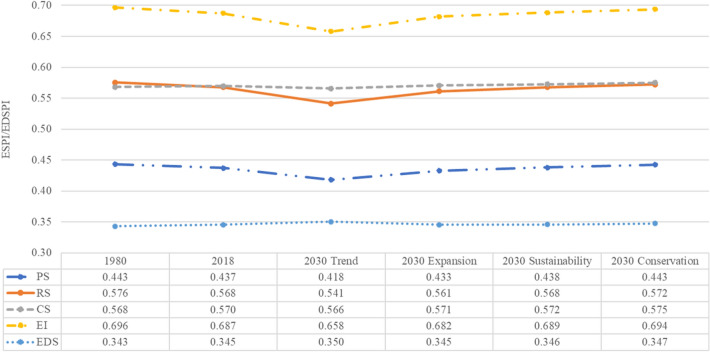
Ecosystem services potential index (ESPI) of provisioning services (ESPI_PS_), regulating services (ESPI_RS_), cultural services (ESPI_CS_), ecological integrity (ESPI_EI_) and ecosystem disservices potential index (EDSPI) dynamic under past (1980), current (2018) and future (2030) land use

The ESPI and EDSPI of different ES and EDS under past (1980), current (2018) and future (2030) land use are shown in Fig. 5. The ESPI of all the ES declined from 1980 to 2018 and will continue to decline until 2030 without sustainable and conservation development strategies (i.e. *Sustainability* and *Conservation* scenarios). The ESPI of all the ES are projected to decrease in the Trend scenario significantly. Similarly, the ESPI_PS_, ESPI_RS_, and ESPI_EI_ are projected to decrease under the *Expansion* scenario, although the ESPI_CS_ will slightly increase. The ESPI will increase under the more sustainable development strategy (i.e. *Sustainability* scenario) from 2018 to 2030, although the ESPI_PS_, ESPI_RS_, and ESPI_EI_ in 2030 will be still lower than those in 1980.

In contrast, the estimation results under the *Conservation* scenario show the implementation of the ecological restoration and protection policy targets would significantly increase the ESPI_PS_, ESPI_RS_, ESPI_CS_, and ESPI_EI_, with the value of ESPI of all the ES reaching the values of 1980. The EDSPI under all future scenarios for 2030 is projected to increase compared to the baseline in 1980. Among these increases, the overall EDSPI of all the EDS is projected to be the largest in the *Trend* scenario because the built-up land delivers the highest EDS score, followed by the Cropland and Woodland, and also, in the *Trend* scenario, the LRB will experience the most extensive urban expansion. EDS increases even under the *Conservation* scenario are due to the combination of the moderate expansion of urban areas (EDS of Floods and Heat island effect), cropland (EDS of Pests and diseases), and afforestation (EDS of Fires).

## Discussion

### Confidence and variability in ES/EDS scores

The uncertainties of expert judgements are often cited as a limitation of the ecosystem services capacity matrix approach (Campagne et al. 2017; Hou et al. 2013; Seppelt et al. 2011). These uncertainties may be sourced from the variability of specific knowledge between experts (Hou et al. 2013) and the variability of experts’ confidence in their scores (Jacobs et al. 2015). However, only a few studies consider the variability and confidence in the analysis with the final scores, although it has been suggested that this analysis of scores should be the norm (Campagne et al. 2020).

Statistical measures such as mean, standard deviation (SD) and confidence score are robust if more than 15 experts are involved in the capacity matrix research (Campagne et al. 2017). We used the SD of the capacity score for each ES and EDS and each ET among the experts to estimate the score variability among the 25 experts (Tables S2 and S3) as this is one useful approach to identify variabilities in scoring agreement between experts (Campagne et al. 2017). Furthermore, the confidence scores were analysed to estimate expert confidence in providing each ES and EDS’s capacity score and each ET (Campagne and Roche 2018; Campagne et al. 2017). The mean SD on all scores provided by 25 experts is only 1.30, and all the SDs in Tables S2 and S3 are less than 2, which means that although the 25 experts were from various institutions and had different disciplinary backgrounds, they all had relatively similar views on the capacities of ES and EDS of different ETs in the LRB. These low levels of SD suggest that the experts who participated in our study are familiar with the overall ecological environment of the LRB, and using the average mean score of the capacity matrix from these experts for determining the ES and EDS of LRB was robust.

According to the confidence scores (Table 2), the experts felt ‘comfortable or moderately comfortable’ on their scores for almost all the ES and EDS. Among the total 616 ES and EDS capacity scores delivered by ETs, the experts had a low level of confidence on only 5 ES and 11 EDS, and most (75%) of the scores with low confidence were from the ES and EDS delivered by ‘Bare land, rock or gravel’, and 25% form the EDS of ‘Human diseases from pathogens’ and ‘Allergens’ delivered from both ‘Industrial area and traffic utilisation’ and ‘Sandy land’. The experts’ unfamiliarity with some EDS was logical as this concept is relatively new, and it was the first time to be applied in the LRB in a research context. Similarly, the contribution of unused lands in ES is often overlooked, and only a few studies focus on this as the unused lands are generally considered to play a negligible role in providing ES (Costanza et al. 1997, 2014; Tolessa et al. 2017). This was also evidenced by our capacity matrix scores, which shows that the average mean score of the ES and EDS delivered by ‘Bare land, rock or gravel’ with low-level confidence was only 0.8. Overall, the results of confidence scores demonstrated that the ES and EDS scores from the experts were reliable.

### Integrating ES and EDS with management strategies: implications for the SDGs

#### Policies related to ES and EDS in the LRB

The Ecological Redline Policy (ERP) in China seeks to sustain critical ecosystem services for social welfare using coordinated planning at a national scale. According to ‘Hebei Province Ecological Protection Redline 2018’ (Hebei Provincial Department of Land and Resources 2018), the basic pattern of the ERP in Hebei is ‘two barriers, two belts and multiple points’. The ‘two barriers’ are the ecological barriers of Yanshan and Taihang Mountain, which provide the main ecological services of soil and water conservation and biodiversity conservation. The ‘two belts’ are windbreak and sand-fixing forest belt of the Bashang Plateau and coastal wetland and shelterbelt. The ‘multi-point’ refers to various ecological protection areas scattered in the plains and mountains. Most of the protected areas are reservoirs, lakes, forests, wetlands and rivers, with functions of flood regulation, runoff regulation, water conservation and biodiversity conservation. The details for policies related to ES and EDS in the different ecological functional zones in the LRB are in the Supplementary Text.

#### Understanding trade-offs among selected SDGs in the LRB

Forests occupy the largest areas in the LRB and represent hotspots for all the ES, meaning that forests should be the land use type of greatest concern when it comes to land management in the LRB. The upstream of the LRB was defined as an important ‘windbreak and sand-fixing area’ to protect Beijing and Tianjin, which are both outside the LRB. The simulation results suggested that the ESPI of all the ES declined from 1980 to 2018 and would continue to decline until 2030 without sustainable and conservation development strategies. The loss of area of forests would be the main driver of such ESPI loss. Under the *Trend*, *Expansion* and *Sustainability* scenarios, the ESPI loss in the LRB would occur due to some areas of forests being replaced by built-up lands, grasslands and croplands. The conversion from forest to built-up lands accounts for the greatest ESPI loss under the *Trend* scenario, while the conversion from forests to the croplands would be the main reason for ESPI loss under the *Expansion* and *Sustainability* scenarios. The results from the modelling under the *Conservation* scenario showed that implementing the ecological restoration and protection policy targets would increase the area of forests, leading to the ESPI increase in the LRB.

Currently, a series of policies promoting afforestation which have been implemented since 2015 in the LRB for sand fixation and biodiversity conservation, such as ‘National Forest Management Planning (2016–2050)’ (State Forestry Administration of China 2016), ‘Land greening planning of Hebei Province (2018–2035)’ (Hebei Provincial Department of Natural Resources 2018), ‘Implementation plan of afforestation in Zhangjiakou city and Chengde Bashang area of Hebei Province’ (State Forestry Administration of China 2019) are encouraging from the perspective of forest area preservation and should continue to be implemented in the future, or even the formulation of more ambitious greening or afforestation policies could be considered in the future. Previous studies in other study areas and the identification of Forest Ecological Zones in the LRB in this study, all have demonstrated that forest plays a vital mitigation role in climate change (Popkin 2019), removing air pollution (Eisenman et al. 2019; Nowak et al. 2006), helping in soil and water conservation (Biao et al. 2010; Zhu et al. 2018) and increasing biodiversity (Sayer et al. 2019). Therefore, these afforestation policies would help achieve SDGs 3 (Good Health and Well-being), 13 (Climate Action), and 15 (Life on land). However, since the ‘Returning Farmland to Forest Program’ is one of the widely implemented measures of afforestation in China (Gao et al. 2020), these afforestation policies likely will, in return, have negative implications for SDGs 1 (No Poverty) and 2 (Zero Hunger). Furthermore, according to the simulation results under future scenarios, with the urban expansion and food demand increases, the built-up land areas and the cropland areas will have a greater demand for other ES, and therefore, increase the pressure on the natural ecosystem.

Synergies and trade-offs exist among most SDGs (Barbier and Burgess 2019). For further understanding, the potential synergies and trade-offs among the SDGs about the future afforestation or urban expansion policies in the LRB, the effects of such policies on selected SDG targets based on indicators and the ES/EDS capacity matrix scores, are highlighted in Table 3. Table 3 shows that the synergies and trade-offs also exist among the SDGs under the afforestation and urban expansion. The ES and EDS are potentially useful for determining the effect of land use changes on SDGs trade-offs and synergies.

**Table 3:** Effects of afforestation and urban expansion on selected SDG targets based on the SDG Indicators and the ES/EDS capacity matrix scores

SDG Target	Target 1.5: Build resilience to environmental, economic and social disasters				Target 2.4: Sustainable food production and resilient agricultural practices	Target 3.9: Reduce illnesses and deaths from hazardous chemicals and pollution	Target 6.3: Improve water quality, wastewater treatment and safe reuse		Target 8.1: Sustainable economic growth	Target 11.5: Reduce the adverse effects of natural disasters	Target 13.1: Strengthen resilience and adaptive capacity to climate-related disasters	Target 13.2: Integrate climate change measures into policy and planning	Target 15.1: Conserve and restore terrestrial and freshwater ecosystems	Target 15.2: End deforestation and restore degraded forests	Target 15.4: Ensure the conservation of mountain ecosystems	Target 15.5: Protect biodiversity and natural habitats	Target 15.8: Prevent invasive alien species on land and in water ecosystems
SDG Indicator	Indicator 1.5.1: Number of deaths, missing persons and directly affected persons attributed to disasters per 100,000 population				Indicator 2.4.1: The proportion of agricultural area under productive and sustainable agriculture	Indicator 3.9.1: Mortality rate attributed to household and ambient air pollution	Indicator 6.3.2: The proportion of bodies of water with good ambient water quality		Indicator 8.1.1: The annual growth rate of real GDP per capita	Indicator 11.5.1: Number of deaths, missing persons and directly affected persons attributed to disasters per 100,000 population	Indicator 13.1.1: Number of deaths, missing persons and directly affected persons attributed to disasters per 100,000 population	Indicator 13.2.2: Total greenhouse gas emissions per year	Indicator 15.1.1: Forest area as a proportion of total land area	Indicator 15.2.1: Progress towards sustainable forest management	Indicator 15.4.2: Mountain Green Cover Index	Indicator 15.5.1: Red List Index	Indicator 15.8.1: The proportion of countries adopting relevant national legislation and adequately resourcing the prevention or control of invasive alien species
Related ES/EDS in this study	Flood protection (RS3)	Fires (EDS4)	Floods (EDS5)	Heat island effect (EDS11)	Crops (PS1) and Livestock (PS2)	Air quality regulation (RS6)	Water purification (RS9)	Droughts (EDS3)	Derived from policy and stakeholder	The same as Indicator 1.5.1		Local and global climate regulation (RS1, RS2)	Directly related to the forest area	Directly related to the forest area	Directly related to the forest area	Biodiversity (EI2)	Invasive species (EDS1)
Afforestation	+	–	+	+	–	+	+	+	–			+	+	+	+	+	–
Urban expansion	/	/	–	–	–	–	/	–	+			–	/	/	/	–	/

“+” refer to the policy has a positive impact on the SDG target in the context of selected ES/EDS, “–” refer to the policy has a negative impact on the SDG target in the context of selected ES/EDS, and “/” refer to the impact cannot be identified based on the ES/EDS capacity matrix scores.

As shown in Table 3, afforestation in the LRB would lead to progress in Targets 1.5, 11.5, and 13.1 by mitigating floods; Target 3.9 by regulating air quality; Target 6.3, 13.2, 15.1, 15.2, and 15.4 by increasing forests area; and Target 15.5 by enhancing biodiversity. Meanwhile, the trade-offs among the SDGs under afforestation are characterised by hindering the progress achieving Target 1.5 since the forests have the high EDSP of Fires (EDS4); Target 2.4 because of the implementation of ‘Returning Farmland to Forest Program’ (Gao et al. 2020); Target 8.1 since the forests impede the expansion of land used for economic development; Target 15.8 as the forests also have the highest EDSP of Invasive species.

Urban expansion in the LRB would contribute to achieving Target 8.1, but it would hinder progress in achieving Target 1.5 by, for example, destroying natural systems and increasing population exposure to natural hazards, although it still depends on the flood protection measures put in place (Zhao et al. 2021); Target 2.4 by reducing the area of cropland (Zhou et al. 2021); Targets 3.9 and 6.3 by increasing pollution; and Targets 11.5, 13.1, 13.2, 15.5 by increasing exposure to natural hazards. Therefore, a sustainable development planning policy for balancing urban expansion and ecological protection in the LRB should be implemented to minimise the trade-offs and maximise the synergies. For example, increasing the number of nature reserves integrated into urban and around the urban area perimeter should be considered in urban development planning. It is also important to set up top–down afforestation and forests restoration policies that encourage local people to protect the biodiversity of forest systems (Zhang et al. 2020), such as the eco-compensation schemes described in the next sub-section.

#### Establishing and implementing cross-regional and trans-provincial eco-compensation schemes for minimising the trade-offs

The trade-offs between rapid economic development, increases in people’s living standards and environmental degradation, which are characteristic of China’s development in general, are also present in the LRB. As one of the ten national innovation demonstration zones for the implementation of the 2030 Agenda, Chengde (of which the administrative boundary accounts for > 60% of the LRB’s area) should focus on improving water and soil conservation function and building the windbreak and sand fixation ecological barrier without harming the local economy (Ministry of Foreign Affairs of the People’s Republic of China 2019). The current trade-offs among SDGs and associated targets in the LRB include: (1) the imposition of limited development in the part of Hebei province in the LRB (e.g. restricting the development of steel industry, banning cage fishing culture of riparian fishermen) to maintain water quantity and quality for Tianjin’s water consumers (Wei et al. 2021); (2) the promotion of afforestation to protect Beijing and Tianjin which are both outside the LRB from wind and sandstorm, achieved by limiting  animal husbandry, planting industry, and mining industry in upstream regions (Li et al. 2017).

Ecological compensation is an important compensatory mechanism to internalise negative environmental externalities for reducing trade-offs, which can be defined as actions that seek to counterbalance ecological values (i.e. natural resources, biodiversity, ecological functions, ecosystem services) that have been or will be impaired by human activities (Arthington et al. 2006; Deal et al. 2012; Shang et al. 2018). After decades of development since the 1970s (Brown and Lant, 1999), ecological compensation is widely accepted as an effective method to protect ecosystems (May et al. 2017). In China, the ‘eco-compensation’—a mechanism aiming to maintain or improve the status of ecosystems by employing economic means to adjust stakeholders’ interests (Shang et al. 2018) has become an important national policy concept to help coordinate regional development relationships, balance development opportunities and ecological protection (Ouyang and Jin, 2017; Zhong et al. 2020). Although the integrated river basin governance perspective helps alleviate trade-offs (Jiang et al. 2021), establishing and effective implementation of sustainable cross-regional and trans-provincial eco-compensation schemes for the LRB is quite challenging.

The system requiring eco-compensation between the LRB and the city of Tianjin is the trans-provincial water supply. The LRB is an important water source for Tianjin, and the water body in the LRB plays an important role in all ES, particularly in terms of freshwater provision (Goal 6: Clean Water and Sanitation). The quantity and quality of water resources in the LRB have profound influences on Tianjin’s socioeconomic development. Over the years, Hebei province has actively taken protective measures, such as the ban on cage fishing in Panjiakou and Daheiting reservoirs and shutting down coal mining and steel enterprises (Wei et al. 2021) and suffering the loss of development opportunities to ensure water safety in Tianjin by investing much labour, material, and financial resources in ecological projects. In 2017, the ‘Upstream and Downstream Eco-compensation Agreement for Water Diversion Project from Luanhe River to Tianjin City’ was signed between Tianjin and Hebei (Xinhua News 2019). However, until June 2019, as the downstream beneficiary (outside of the LRB basin), Tianjin has only paid the amount of 300 million RMB to Hebei for the improvement of water quality, which is a very small compensation compared to the benefits Hebei has brought to Tianjin (Xinhua News 2019; Yang et al. 2020).

The biggest challenge in the policymaking of a trans-provincial compensation mechanism was the divergence of interests between provinces. First, the two sides had greater differences in the premise of compensation. Tianjin insisted that the Panjiakou and Daheiting Reservoirs should be classified as a water source protection zone. However, once the water source protected zone was delineated, cage fishing in the reservoir area had to be banned and farming and human activities in the reservoir area limited. Due to the difficulty of resettlement of a large number of aquaculture households, Hebei was opposed to this plan. Second, there was no initial agreement on the purpose and content of establishing an ecological compensation mechanism. Tianjin was willing to give financial compensation to the water source protection project implemented in Hebei. However, Hebei wanted to obtain more comprehensive compensation because this was not only about the cost for water source protection and water pollution control but also about addressing poverty due to the development opportunities lost for protecting water sources. Third, at present, the recognition of existing eco-compensation methods in Tianjin and Hebei seems to be limited to the recognition of existing ecological compensation methods—the downstream government fiscal funds only compensate the upstream areas. The behaviours from enterprises, society and the private sector have not been included in the compensation scope, so the actual compensation scale may be underestimated. It also should be noted that although the LRB is an important water source for Tianjin, Tianjin has already created a multi-source water supply system after the opening of the middle water supply route of China’s South-to-North Water Transfer Project in December 2014 (Zhang et al. 2018). It is possible that, in the future, Tianjin may stop compensating Hebei if it sources another more competitive provider for their drinking water needs (e.g. more water from the South-to-North Water Transfer Project). This potential scenario means that Hebei province may take the ‘risk’ in making all the changes based on an eco-compensation agreement. The environmental condition and socioeconomic activities that previously occurred in the LRB, as mentioned above, are unlikely to return. Therefore, there is a need for an effective information-sharing mechanism among multiple stakeholders and regional governments to establish the long-term guaranteed trans-provincial eco-compensation standard by combining gross ecosystem product and full cost accounting under the guidance of the national government (Fang et al. 2021).

Moreover, a sustainable cross-regional ecological compensation mechanism for balancing the development opportunities and ecological protection between upstream and downstream should be refined and effectively implemented. The animal husbandry, planting industry, and mining industry in upstream regions have been limited to promote afforestation and protect downstream regions from wind and sandstorms. This inevitably impacts the livelihoods of farmers and herdsmen, and as a result, affects the economic development of upstream regions (Goal 2: Zero Hunger and Goal 8: Decent Work and Economic Growth). A sustainable trade-off needs to be preserved to maintain other PS such as food which remains important given the rapid urban extension in the region (Goal 11: Sustainable Cities and Communities). For this, a sustainable ecological compensation mechanism between upstream and downstream regions for increasing financial transfer payments to upstream ecological protection areas should be refined and effectively implemented. Furthermore, since the forests have the highest EDSP of fires, a strict forest fires prevention policy is also necessary.

### Limitations and uncertainties of the research and future directions

The ES and EDS capacity matrix depends on expert knowledge, and the results are therefore inevitably relatively subjective and introduce uncertainties (Burkhard et al. 2009, 2012; Campagne et al. 2020, 2018). Nevertheless, recent studies have compared experts-based matrix scores with quantitative estimates (e.g. Ma et al. (2019); Roche and Campagne (2019)) and demonstrated that the more complex ES and EDS assessment approaches do not necessarily deliver more robust results than those expert scoring (Campagne et al. 2020; Jacobs and Burkhard, 2017). In this study, we followed methods demonstrated to minimise the uncertainties of expert judgements (Campagne et al. 2017, 2020), such as identifying the appropriate size of a qualified expert panel and applying confidence scores and SD. According to Campagne et al. (2020), the selection and the number of experts in the panel, and the elicitation methods used to produce the estimates is extremely important for the robustness of the results. A robust and adaptable standard for selecting and communicating with the expert panel is required in future work.

Additionally, the ES and EDS capacity matrix approach is mainly based on spatial units as ET (land use) categories, although land use categories are considered as important proxies for ES and EDS (Hasan et al. 2020; Lawler et al. 2014; Metzger et al. 2006; Mouchet et al. 2017). In this study, we considered the land management at the river basin scale to a certain extent. The future dynamics of ETs in the LRB under different scenarios in 2030 (Table 1) was derived from the simulated land systems based on the CLUMondo model by Xu et al. (2021). These future land systems have considered not only the land use and cover but also the livestock and agricultural intensity data which represent important characteristics of land management and farming systems at the river basin scale. Nevertheless, in this study, land use alone lacks local or pilot-scale information regarding important components of ecosystem conditions that support ES capacities, such as soil type and quality, water availability, geomorphology, which will be affected by land management and vary in space and time. The spatiotemporal invariance of the capacity matrix results in the distant areas with the same ET to have the same scores without accounting for their specificity (Campagne et al. 2020; Jacobs et al. 2015). The local or regional land management practices, such as the policies implemented in different ecological functional zones in the LRB, would change the ecosystem conditions or even the socioeconomic condition (e.g. human population density or economy).

In addition, although the ESPI and EDSPI are straightforward and easily applied for indicating the changes of the potential of a region to deliver ecosystem services under past, current and future land use in the LRB, they lack consideration of temporal and spatial variations at different levels of scale. In this study, using the EDSPI, we consider each ET provides the value of EDS equally at the river basin scale from 1980 to 2030. However, the provision and value of EDS might also vary in time and space (Dobbs et al. 2014; Linden et al. 2019). Some local scale EDS (e.g. dangerous plants/animals) may not be problematic if there is no direct contact between people and these disservices at the river basin scale. Such limitations also result in little difference between the ESPI and EDSPI values under different scenarios. The normalised indexes, ESPI and EDSPI, have successfully indicated the long-term trend of ES and EDS changes from 1980 to 2030 at the river basin scale. Nevertheless, the ES and EDS dynamic due to the area change of ETs or land management activities at a local scale is not captured by the river basin scale indexes like ESPI and EDSPI. In future studies, additional spatial analysis can be factored in, or spatial invariance (e.g. regional variation) can be considered in the ETs lists in the matrix. For example, the experts could also be asked to provide ranges of ES and EDS values depending on different ecosystem states, locations (e.g. upstream, midstream and downstream of the river basin), or under different land management practices (e.g. poor, average, good). In addition, more studies to understand the localised weighted coefficients might be useful to realise an accurate assessment of the ES and EDS for the study area.

## Conclusions

In this study, an integrated research framework for evaluating the ES and EDS, identifying hotspots of critical ES and EDS, and understanding the relationship between ES and EDS and river basin management under past, current and future land use scenarios is presented. Applying the stakeholder’s participatory ecosystem service capacity matrix to the LRB, the ecosystem services and SDGs trade-offs and synergies are investigated to provide policy recommendations.

The results show that the LRB is a diverse landscape with hotspots for ES and EDS, defined as six ecological functional zones. However, the ecosystems in the LRB are threatened in the future because of changes in land use without sustainable and conservation development strategies, mainly reflected in the decreases of ES and increases of EDS. The ES and EDS are a useful bridge to understand the effect of land use on the SDGs and associated targets and indicators. This study demonstrated that the impacts of ES and EDS changes would not be homogeneous in the landscape. These changes will positively and negatively affect different SDG targets in different locations of the LRB. For maximising synergies and reducing trade-offs between SDGs and associated targets on the sub-national scale, policy recommendations such as establishing and implementing sustainable environmental protection policies and cross-regional and trans-provincial eco-compensation schemes are suggested. The integrated research framework simulating the ES and EDS under past, current and future development scenarios, especially taking into account stakeholders’ opinions and various levels of policy, could be used for identifying hotspots and coldspots of ES and EDS at the river basin scale, providing valuable stakeholders’ perspectives for environmental management, and help establish and implement the policy for minimising the trade-offs and maximising the synergies between SDGs and targets.

## Supplementary Information

Below is the link to the electronic supplementary material.

Supplementary file1 (DOCX 203 KB)
