# Supplementary material for: Ecosystem services and disservices in the Luanhe River Basin in China under past, current and future land uses: implications for the sustainable development goals
Source: Sustain Sci. 2022 Jan 8;17(4):1347–64. doi: 10.1007/s11625-021-01078-8 (PMC8741573; doi:10.1007/s11625-021-01078-8)
Supplement: Supplementary file 1 — Supplementary file1 (DOCX 203 KB) [file 11625_2021_1078_MOESM1_ESM.docx]

*Sustainability Science*

Supporting Information for

**Ecosystem services and disservices in the Luanhe River Basin in China under past, current and future land uses: implications for the Sustainable Development Goals**

Jiren Xu^1^, Brian Barrett^2^, Fabrice G. Renaud^1^

^1^ School of Interdisciplinary Studies, University of Glasgow, Dumfries, DG1 4ZL UK

^2^ School of Geographical & Earth Sciences, University of Glasgow, Glasgow, G12 8QQ UK

^*^ Corresponding author. Email address: [jiren.xu@glasgow.ac.uk](mailto:jiren.xu@glasgow.ac.uk)

Tel: +44 (0)1387702091

**Contents of this file**

Table S1 - 3, and Supplementary Text

**Introduction**

This file of supplemental material contains three Tables and Supplementary Text. Table S1 shows the six ecological function zones in the LRB. Table S2 shows the standard deviation (SD) of the capacity scores for each ecosystem service and each ecosystem type among the 25 experts. Table S3 shows the standard deviation (SD) of the capacity scores for each ecosystem disservices and each ecosystem type among the 25 experts. The Supplementary Text including the introduction to the land system simulation workflow and introduction to the six ecological zones.

**Table S1** Six ecological function zones in the LRB.


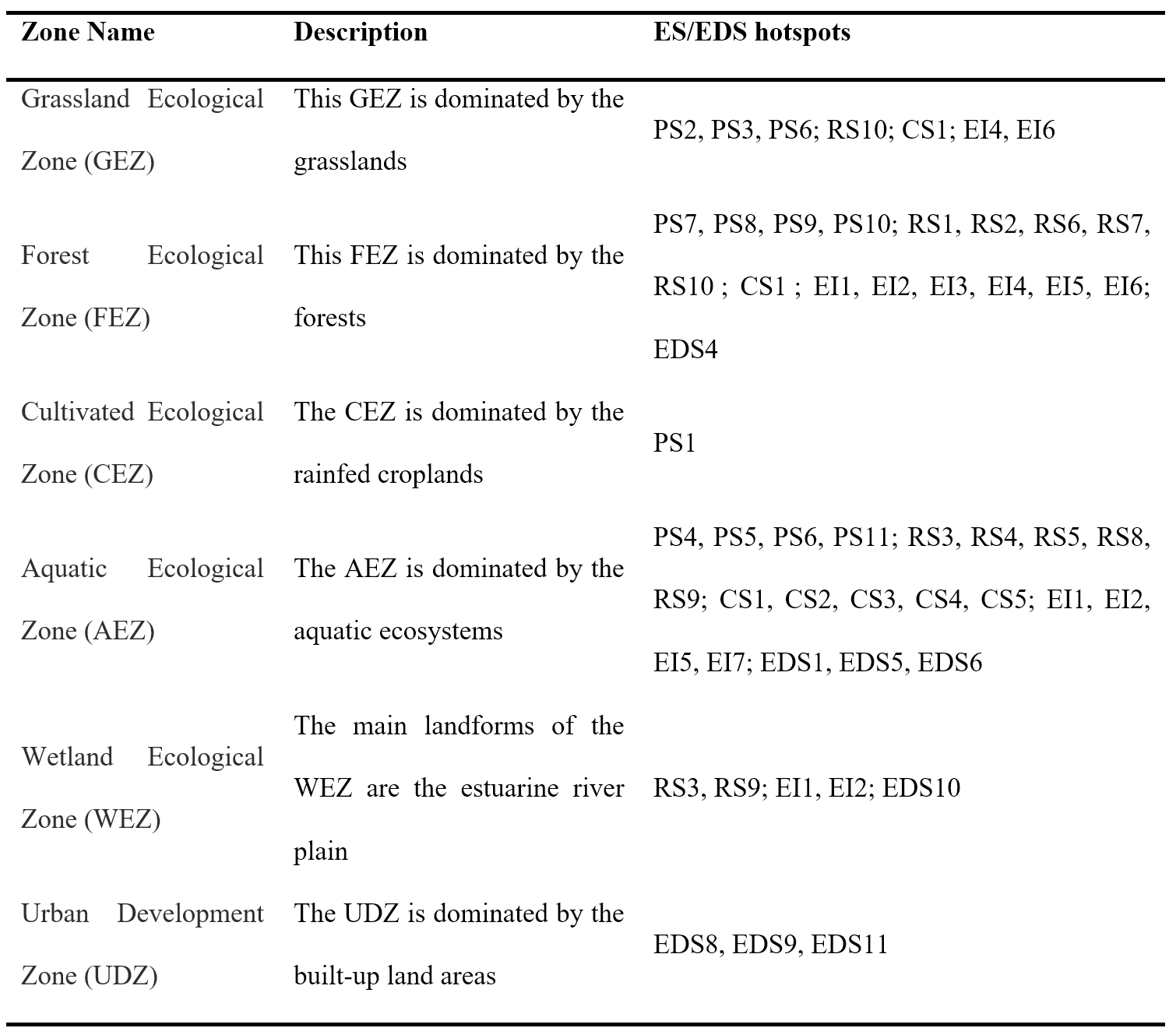


**Table S2** The standard deviation (SD) of the capacity scores for each ecosystem service and ecosystem type among the 25 experts.

|  |  | **Provisioning Services (PS)** | | | | | | | | | | | **Regulating services (RS)** | | | | | | | | | | **Regulating services (RS)** | | | | | **Ecological Integrity (EI)** | | | | | |
| --- | --- | --- | --- | --- | --- | --- | --- | --- | --- | --- | --- | --- | --- | --- | --- | --- | --- | --- | --- | --- | --- | --- | --- | --- | --- | --- | --- | --- | --- | --- | --- | --- | --- |
|  |  | **Crops** | **Livestock** | **Fodder** | **Capture fisheries** | **Aquaculture** | **Wild foods** | **Timber** | **Wood fuel** | **Energy (biomass)** | **Biochemicals and medicine** | **Freshwater** | **Local climate regulation** | **Global climate regulation** | **Flood protection** | **Fire protection** | **Groundwater recharge** | **Air quality regulation** | **Erosion regulation** | **Nutrient regulation** | **Water purification** | **Pollination** | **Aesthetic** | **Existence and bequest** | **Knowledge and education** | **Emblematic and symbolic** | **Physical and experiential interactions** | **Abiotic heterogeneity** | **Biodiversity** | **exergy capture** | **Reduction of nutrient loss** | **Storage capacity** | **Metabolic efficiency** |
| **Cropland** | **Irrigated croplands** | 0.40 | 1.64 | 1.43 | 1.21 | 1.38 | 1.07 | 0.64 | 1.08 | 1.73 | 1.16 | 1.52 | 1.13 | 1.49 | 1.79 | 2.01 | 1.73 | 1.56 | 1.55 | 1.38 | 1.61 | 1.30 | 1.36 | 1.50 | 1.40 | 1.38 | 1.29 | 1.49 | 1.58 | 1.27 | 1.38 | 1.32 | 1.21 |
|  | **Rainfed croplands** | 0.63 | 1.20 | 0.90 | 1.12 | 1.21 | 1.40 | 0.99 | 1.70 | 1.52 | 1.44 | 1.02 | 1.39 | 1.62 | 1.74 | 1.35 | 1.41 | 1.38 | 1.35 | 1.50 | 1.37 | 1.08 | 1.08 | 1.23 | 1.37 | 1.26 | 1.28 | 1.35 | 1.53 | 1.12 | 1.42 | 1.25 | 1.39 |
| **Woodland** | **Forest** | 1.59 | 1.35 | 1.61 | 1.12 | 1.12 | 1.65 | 0.37 | 0.65 | 1.24 | 1.26 | 1.52 | 0.56 | 0.95 | 1.32 | 1.46 | 1.08 | 1.21 | 0.91 | 1.08 | 1.16 | 1.16 | 1.23 | 1.77 | 1.32 | 1.50 | 1.35 | 0.68 | 0.63 | 1.11 | 0.83 | 0.74 | 1.31 |
|  | **Nursery and orchard** | 1.81 | 1.39 | 1.25 | 1.00 | 1.00 | 1.63 | 1.36 | 1.04 | 1.53 | 1.56 | 1.28 | 0.81 | 1.16 | 1.38 | 1.33 | 0.94 | 1.02 | 1.46 | 1.15 | 1.22 | 1.10 | 1.35 | 1.52 | 1.42 | 1.43 | 1.25 | 0.58 | 1.07 | 1.11 | 1.02 | 0.94 | 1.38 |
| **Grassland** | **Grassland** | 1.70 | 1.12 | 1.04 | 1.61 | 1.41 | 1.69 | 1.51 | 1.46 | 1.50 | 1.35 | 1.34 | 1.38 | 1.34 | 1.32 | 1.47 | 1.29 | 1.37 | 1.22 | 1.31 | 1.27 | 1.64 | 0.93 | 1.87 | 1.54 | 1.76 | 0.99 | 1.08 | 0.93 | 1.00 | 1.07 | 0.92 | 1.54 |
| **Water body** | **Stream and river** | 1.88 | 1.90 | 1.71 | 1.52 | 1.58 | 1.83 | 1.48 | 1.42 | 1.52 | 1.58 | 1.08 | 1.34 | 1.30 | 1.56 | 1.47 | 1.35 | 1.58 | 1.46 | 1.75 | 1.62 | 1.02 | 1.44 | 1.91 | 1.57 | 1.93 | 1.33 | 1.38 | 1.50 | 1.33 | 1.50 | 1.64 | 1.53 |
|  | **Lakes** | 1.99 | 1.78 | 1.71 | 1.71 | 1.17 | 1.61 | 1.49 | 1.44 | 1.51 | 1.51 | 1.04 | 0.93 | 1.23 | 1.32 | 1.28 | 1.00 | 1.57 | 1.66 | 1.73 | 1.43 | 1.00 | 0.77 | 1.83 | 1.55 | 1.69 | 0.76 | 0.54 | 0.87 | 1.42 | 1.29 | 1.10 | 1.36 |
|  | **Reservoirs and ponds** | 1.18 | 1.00 | 1.70 | 1.40 | 1.20 | 1.52 | 1.54 | 1.39 | 1.64 | 1.49 | 1.12 | 1.00 | 1.29 | 0.91 | 1.21 | 1.03 | 1.58 | 1.51 | 1.68 | 1.53 | 1.00 | 1.41 | 1.83 | 1.68 | 1.83 | 1.22 | 1.09 | 1.12 | 1.40 | 1.24 | 1.28 | 1.26 |
|  | **Beach and shore** | 1.79 | 1.87 | 1.57 | 1.70 | 1.76 | 1.33 | 1.46 | 1.32 | 1.29 | 1.38 | 1.65 | 1.38 | 1.48 | 1.34 | 1.70 | 1.38 | 1.64 | 1.58 | 1.34 | 1.45 | 1.19 | 1.22 | 1.80 | 1.41 | 1.71 | 1.56 | 1.08 | 1.08 | 1.14 | 1.21 | 1.15 | 1.44 |
| **Built-up land** | **Residential/commercial areas** | 1.04 | 1.38 | 1.08 | 1.04 | 0.91 | 0.81 | 1.16 | 1.23 | 1.12 | 1.05 | 0.87 | 0.91 | 1.18 | 0.87 | 1.48 | 0.92 | 1.12 | 1.73 | 0.77 | 1.00 | 0.76 | 1.81 | 1.83 | 1.68 | 1.67 | 1.45 | 1.26 | 0.78 | 0.79 | 0.98 | 1.03 | 1.09 |
|  | **Industrial area** | 0.75 | 0.87 | 0.75 | 0.88 | 0.72 | 0.96 | 0.96 | 1.23 | 1.07 | 1.59 | 0.91 | 0.87 | 1.22 | 0.76 | 1.27 | 0.86 | 1.16 | 1.78 | 0.76 | 0.75 | 0.63 | 0.91 | 1.76 | 1.49 | 1.71 | 1.16 | 1.44 | 0.71 | 0.92 | 0.96 | 0.95 | 1.08 |
| **Unused land** | **Sandy land** | 1.01 | 0.98 | 0.99 | 0.84 | 0.72 | 1.28 | 1.08 | 1.12 | 1.51 | 1.61 | 0.86 | 0.95 | 0.98 | 1.60 | 1.83 | 1.46 | 1.08 | 1.34 | 0.95 | 1.22 | 0.97 | 1.56 | 1.56 | 1.55 | 1.44 | 1.36 | 1.51 | 1.08 | 0.92 | 0.99 | 1.05 | 0.78 |
|  | **Swamp** | 1.71 | 1.56 | 1.45 | 1.61 | 1.38 | 1.64 | 1.45 | 1.44 | 1.46 | 1.64 | 1.45 | 1.59 | 1.42 | 1.81 | 1.83 | 1.50 | 1.79 | 1.41 | 1.53 | 1.40 | 1.32 | 1.33 | 1.38 | 1.39 | 1.23 | 1.31 | 1.24 | 1.25 | 1.66 | 1.49 | 1.47 | 1.70 |
|  | **Bare land, rock or gravel** | 1.04 | 0.96 | 0.77 | 0.75 | 0.72 | 1.54 | 0.99 | 0.90 | 1.00 | 0.87 | 0.71 | 1.19 | 1.13 | 1.38 | 1.40 | 1.22 | 0.95 | 0.81 | 0.82 | 0.87 | 1.04 | 1.08 | 1.14 | 1.27 | 1.02 | 1.05 | 1.37 | 1.38 | 1.05 | 0.99 | 1.12 | 1.06 |

**Table S3** The standard deviation (SD) of the capacity scores for each ecosystem disservices and ecosystem type among the 25 experts.

|  |  | **Ecosystem Disservices (EDS)** | | | | | | | | | | | |
| --- | --- | --- | --- | --- | --- | --- | --- | --- | --- | --- | --- | --- | --- |
|  |  | **Biotic water flows** | **Invasive species** | **Pests and diseases** | **Droughts** | **Fires** | **Floods** | **Erosion and siltation** | **Leaching of nutrients** | **Human diseases from pathogens** | **Allergens** | **Dangerous plants and animals** | **Heat island effect** |
| **Cropland** | **Irrigated croplands** | 1.41 | 1.45 | 1.42 | 1.62 | 1.12 | 1.32 | 1.32 | 1.19 | 1.27 | 1.00 | 1.03 | 0.77 |
|  | **Rainfed croplands** | 1.12 | 1.34 | 1.19 | 1.50 | 1.56 | 1.19 | 1.31 | 1.42 | 1.08 | 1.00 | 1.04 | 1.51 |
| **Woodland** | **Forest** | 1.17 | 1.37 | 1.14 | 1.63 | 1.01 | 1.00 | 1.16 | 1.44 | 1.05 | 1.47 | 1.23 | 1.08 |
|  | **Nursery and orchard** | 1.19 | 1.22 | 1.05 | 1.56 | 1.10 | 1.05 | 1.09 | 1.26 | 1.07 | 1.18 | 1.40 | 1.29 |
| **Grassland** | **Grassland** | 1.46 | 1.27 | 1.12 | 1.74 | 1.57 | 1.05 | 1.16 | 1.35 | 1.11 | 1.23 | 1.33 | 1.16 |
| **Water body** | **Stream and river** | 1.66 | 1.35 | 1.50 | 1.41 | 0.79 | 1.88 | 1.82 | 1.32 | 1.25 | 1.10 | 1.39 | 0.87 |
|  | **Lakes** | 1.53 | 1.41 | 1.63 | 1.44 | 0.83 | 1.87 | 1.72 | 1.49 | 1.24 | 1.22 | 1.39 | 0.82 |
|  | **Reservoirs and ponds** | 1.53 | 1.76 | 1.72 | 1.41 | 0.71 | 2.02 | 2.01 | 1.63 | 1.42 | 1.61 | 1.55 | 0.92 |
|  | **Beach and shore** | 1.11 | 1.15 | 1.21 | 1.26 | 0.91 | 1.81 | 1.42 | 1.46 | 1.47 | 1.76 | 1.49 | 0.63 |
| **Built-up land** | **Residential/commercial areas** | 0.99 | 1.69 | 0.96 | 1.66 | 1.59 | 1.77 | 1.86 | 1.41 | 1.89 | 2.02 | 1.29 | 1.45 |
|  | **Industrial area** | 0.87 | 1.49 | 0.82 | 1.81 | 1.56 | 1.88 | 2.10 | 1.36 | 1.79 | 1.81 | 1.38 | 1.45 |
| **Unused land** | **Sandy land** | 1.04 | 1.26 | 0.76 | 2.12 | 1.94 | 1.02 | 2.13 | 1.58 | 0.87 | 1.02 | 0.84 | 1.90 |
|  | **Swamp** | 1.28 | 1.12 | 1.05 | 1.46 | 1.04 | 1.68 | 1.50 | 1.41 | 1.55 | 1.78 | 1.62 | 0.63 |
|  | **Bare land, rock or gravel** | 0.94 | 0.90 | 0.92 | 1.31 | 1.66 | 0.71 | 1.22 | 1.03 | 0.94 | 0.91 | 1.15 | 0.71 |

**Supplementary Text for ‘Ecosystem services and disservices in the Luanhe River Basin in China under past, current and future land uses: implications for the Sustainable Development Goals’**

**Introduction to the *land system simulation workflow***

The simulation was conducted in three steps. First, land systems of the LRB in the years 2000 and 2015 were mapped by integrating different datasets related to human-environment attributes. Then, the relationship between the land systems and local explanatory factors was calculated for the initial year (2000). Second, the CLUMondo model (Van Asselen and Verburg, 2013) was parameterised and calibrated based on the 2015 land systems map. Finally, changes in the land systems from 2015 to 2030 were simulated under different scenarios, including alternative demands for commodities and services. They represented different pathways on managing LRB’s land resources. More details are available in Xu et al. (2021)

**Introduction to *six ecological functional zones***

Six ecological functional zones were defined as follow:

Grassland Ecological Zone (GEZ) (I) includes hotspots of 6 individual types of ES: livestock, fodder, and wild foods (PS); pollination (RS); aesthetic value (CS); reduction of nutrient loss and metabolic efficiency (EI). Forest Ecological Zone (FEZ) (II) has various ES hotspots types belonging to all ES categories. However, the FEZ is also the hotspot of fires of EDS. Cultivated Ecological Zone (CEZ) (III) is delivering the ES of crops provisioning. The city sprawling and developing residential areas will progressively occupy this zone and decrease the services provided by this region’s ecosystems. Aquatic Ecological Zone (AEZ) (IV) has various ES hotspots types belonging to all ES categories. However, invasive species, floods, and erosion and siltation are the main EDSs in this zone. Wetland Ecological Zone (WEZ) (V) provides important ES of flood protection and water purification (RS), abiotic heterogeneity and biodiversity (EI). The only major EDS is the risk of ‘dangerous or poisonous plants and animals’. Urban Development Zone (UDZ) (VI): The zone of the built-up land areas represent coldspots of PS, RS and EI, and are the hotspots of EDSs in the LRB. With the urban expansion, this zone will have a greater demand for other ESs in the future and will therefore increase the pressure on the natural ecosystems in the rest of the LRB.

Grassland Ecological Zone (GEZ) (I): This zone belongs principally to the south edge of the Inner Mongolia plateau and is dominated by the grassland ecosystem. The GEZ includes hotspots of 6 individual types of ES: livestock, fodder, and wild foods (PS); pollination (RS); aesthetic value (CS); reduction of nutrient loss and metabolic efficiency (EI).

Forest Ecological Zone (FEZ) (II): The zone is mainly located in the north-eastern part of Hebei province, bordering the Bashang Plateau in the north, and the Hebei Plain in the south is dominated by forests. The FEZ has a variety of ES hotspots types belonging to all ES categories: timber, wood fuel, energy (biomass), biochemicals and medicine (ES); local climate regulation, global climate regulation, air quality regulation, erosion regulation, and pollination (RS); aesthetic (CS); abiotic heterogeneity, biodiversity, exergy capture, reduction of nutrient loss, storage capacity, and metabolic efficiency (EI). However, the FEZ is also the hotspot of fires of EDS.

Cultivated Ecological Zone (CEZ) (III): The CEZ is dominated by the rainfed croplands surrounding the urban areas and mainly concentrated in the north-western and south-eastern regions of the LRB, which is delivering the ES of crops provisioning. The city sprawling and developing residential areas will progressively occupy this zone and decrease the services provided by this region’s ecosystems.

Aquatic Ecological Zone (AEZ) (IV): Aquatic ecosystems such as lakes, rivers, and reservoirs are dominant ecosystem types in the AEZ. The AEZ has a variety of ES hotspots types belonging to all ES categories: capture fisheries, aquaculture, wild foods, and freshwater (ES); flood protection, fire protection, groundwater recharge, nutrient regulation and water purification (RS); aesthetic, existence and bequest, knowledge and education, emblematic and symbolic, and physical and experiential interactions (CS); abiotic heterogeneity, biodiversity, storage capacity and biotic water flows (EI). However, invasive species, floods, and erosion and siltation are the main EDS in this zone. It should be noted that the water supply for regional residents’ consumption and livelihoods depends on rivers and lakes. Still, the great demand for CS, such as entertainment and leisure activities of regional residents, also relied on conserving the water bodies in this zone. However, since the AEZ is interacting with farmlands and urban areas, urban domestic sewage discharge, industrial wastewater, and agricultural non-point source pollution are major pressures threatening the ES in the AEZ.

Wetland Ecological Zone (WEZ) (V): The main landforms of the WEZ are the estuarine river plain in the south-eastern region and the swamps in the north-western regions. Beach and shores, and swamps are the dominant ecosystem types in this zone. Although the area of WEZ only accounts for less than 2% of the whole LRB, this zone provides important ES of flood protection and water purification (RS), abiotic heterogeneity and biodiversity (EI). The only major EDS is the risk of ‘dangerous or poisonous plants and animals’.

Urban Development Zone (UDZ) (VI): The zone of the built-up land areas represent coldspots of PS, RS and EI, and are the hotspots of EDSs in the LRB. With the urban expansion, this zone will have a greater demand for other ESs in the future and will increase the pressure on the natural ecosystems in the rest of the LRB. Sustainable development planning for balancing the urban expansion and ecological protection in the LRB should be prioritised to avoid trade-offs between ES provision in the basin, with implications for the SDGs.

**Policies related to ecosystem services and disservices in the LRB**

The Ecological Redline Policy (ERP) in China seeks to sustain critical ecosystem services for social welfare using coordinated planning at a national scale. According to ‘Hebei Province Ecological Protection Redline’ (Hebei Provincial Department of Land and Resources, 2018), the basic pattern of the ERP in Hebei is ‘two barriers, two belts and multiple points’. The ‘two barriers’ are the ecological barriers of Yanshan and Taihang Mountain, which provide the main ecological services of soil and water conservation and biodiversity conservation. The ‘two belts’ are windbreak and sand-fixing forest belt of the Bashang Plateau and coastal wetland and coastal shelterbelt. The ‘multi-point’ refers to various ecological protection areas scattered in the plains and mountains. Most of the protected areas are reservoirs, lakes, forests, wetlands and rivers, with functions of flood regulation, runoff regulation, water conservation and biodiversity conservation.

According to the ‘General Land Use Planning in Hebei Province (2006-2020)’ (Hebei Provincial Department of Land and Resources, 2010), ‘Land greening planning of Hebei Province (2018-2035)’ (Hebei Provincial Department of Natural Resources, 2018) and ‘Implementation plan of afforestation in Zhangjiakou city and Chengde Bashang area of Hebei Province (State Forestry Administration of China, 2019)’, the GEZ is located in the ‘Windbreak and sand-fixing forest belt of Bashang Plateau - the priority eco-regions for biodiversity conservation of Hilly and Plateau areas’. The policy focuses on reducing wind hazards, sand fixation and water and soil conservation. The policy calls for building shelterbelts, returning farmland to forests and grasslands appropriately, and residence emigration. The policy also adjusts the structure of agriculture and controls the amount of livestock on grasslands. Under this policy, the grassland quality will be protected, and the capacity to enhance biodiversity will be increased. Although the capacity of providing livestock and fodder is negatively affected due to the amount of livestock on grasslands being controlled, in the short term, this could contribute to a more sustainable capacity of PS of grassland in the long term.

The FEZ is mainly located in the ‘Priority eco-regions for biodiversity conservation of Taihang Mountain area in West Hebei, Hilly area in North Hebei’, and partly located in the ‘Priority eco-regions for biodiversity conservation of Hilly and Plateau areas’. As for the ‘Priority eco-regions for biodiversity conservation of Taihang Mountain’, the policy calls for strengthening the forests for water and soil conservation, implementing the Beijing-Tianjin sandstorm source control project, returning farmland to forests, and protecting natural forests. The policy intensifies comprehensive efforts to control the surrounding areas of reservoirs and the upper reaches of rivers, strengthen management over the exploitation of mineral resources, implement comprehensive management of the mining environment, and constantly improve the ecological environment. The policy also strengthens the management and protection of nature reserves to protect and restore biodiversity. These policies should contribute to maintain or even strengthen the overall capacity of ES, since the forests are considered to be significantly important for PS, RS, CS and EI. Furthermore, the implementation of strengthening soil and water conservation in forests would be helpful to decrease the EDSP of fires.

As for the AEZ, the policy of ‘priority eco-regions for biodiversity conservation of Inland River and Lake Area’ (Hebei Provincial Department of Land and Resources, 2010) and the policy of ecological redline of ‘Riparian ecological redline areas of river and lake on Hebei plain’ both aim at improving the ecological environment, regulating water resources, and protecting the aquatic ecosystems (e.g. habitats of aquatic wildlife). The policy strictly controls polluting enterprises’ activities (e.g. coal mining, steel industry, and cage fishing), raising standards for sewage treatment plants, ensuring that sewage meets discharge standards, and reducing pollution from tourist waste. The policies would improve the overall capacity of ES of LRB since the waterbody is significantly important for PS, RS, CS and EI. Such as the PS of freshwater and RS of fire protection, groundwater recharge would benefit from the policy of regulating water resources, and capacity of water purification and the CS of aesthetic, emblematic and symbolic, and physical and experiential interactions would also be increased due to the better water quality and aquatic ecosystem environment. However, it should be noticed that these policies would be potentially harmful to the capacity of the PS of aquaculture. For example, the banning of cage fishing in the midstream of LRB (e.g. Panjiakou reservoir and Daheitting reservoir) during the past few years for preserving the ecological functions of freshwater provision have severely limited the development of local aquaculture (Kang et al., 2020; Wei et al., 2021).

It should be noticed that these policies have also been included in the scenarios (Xu et al., 2021), which have been applied in this research. For example, the ‘General Land Use Planning in Hebei Province (2006-2020)’, ‘Land greening planning of Hebei Province (2018-2035)’ (Hebei Provincial Department of Natural Resources, 2018) and ‘Implementation plan of afforestation in Zhangjiakou city and Chengde Bashang area of Hebei Province (State Forestry Administration of China, 2019)’ have been included in the *Sustainability* and *Conservation* scenarios. The demands for the area of different land-use types in the future were derived based on the data published in these documents.

## **References**

Hebei Provincial Department of Land and Resources, 2010. General Land Use Planning in Hebei Province (2006-2020) (in Chinese).

Hebei Provincial Department of Land and Resources, 2018. Hebei Province Ecological Protection Redline.

Hebei Provincial Department of Natural Resources, 2018. Land greening planning of Hebei Province (2018-2035) (in Chinese).

Kang, G., Yin, J., Cui, N., Ding, H., Wang, S., Wang, Y. and Qi, Z., 2020. The Long-Term and Retention Impacts of the Intervention Policy for Cage Aquaculture on the Reservoir Water Qualities in Northern China. Water, 12(12): 3325.

State Forestry Administration of China, 2019. Implementation plan of afforestation in Zhangjiakou city and Chengde Bashang area of Hebei Province (in Chinese). China Forestry Publishing House, Beijing, China.

Van Asselen, S. and Verburg, P.H., 2013. Land cover change or land‐use intensification: simulating land system change with a global‐scale land change model. Global change biology, 19(12): 3648-3667.

Wei, M., Huang, S., Li, L., Zhang, T., Akram, W., Khatoon, Z. and Renaud, F.G., 2021. Evolution of water quality and biota in the Panjiakou Reservoir, China as a consequence of social and economic development: implications for synergies and trade-offs between Sustainable Development Goals. Sustainability Science, forthcoming.

Xu, J., Renaud, F.G. and Barrett, B., 2021. Modelling land system evolution and dynamics of terrestrial carbon stocks in the Luanhe River Basin, China: a scenario analysis of trade-offs and synergies between sustainable development goals. Sustainability Science, 10.1007/s11625-021-01004-y.
